# Gas Chromatography-Mass Spectrometry Based Midgut Metabolomics Reveals the Metabolic Perturbations under NaF Stress in *Bombyx mori*

**DOI:** 10.3390/insects11010017

**Published:** 2019-12-24

**Authors:** Gang Li, Xiao Zhang, Heying Qian, Mingzhu Liu, Guodong Zhao, Anying Xu

**Affiliations:** 1 The Sericultural Research Institute, Jiangsu University of Science and Technology, Zhenjiang 212003, China; 2 The key Laboratory of silkworm and mulberry genetic improvement, Ministry of Agriculture, Chinese Academy of Agricultural Science, Zhenjiang 212018, China

**Keywords:** fluoride tolerance, untargeted metabolomics, silkworm midgut, differential metabolites, metabolic pathways

## Abstract

Fluoride tolerance is an important economic trait in sericulture, especially in some industrial development regions. Analyses of physiological changes involving structural damage to the insect body and molecular analyses of some related genes have focused on this area; however, the changes that occur at the metabolic level of silkworms after eating fluoride-contaminated mulberry leaves remain unclear. Here, metabonomic analysis was conducted using gas chromatography-mass spectrometry (GC-MS) to analyze the changes in midgut tissue after NaF stress using silkworm strains 733xin (susceptible stain) and T6 (strain resistant to fluoride), which were previously reported by our laboratory. Differential metabolomics analysis showed that both T6 and 733xin strains displayed complex responses after exposure to 200 mg/kg NaF. The purine metabolism and arginine and proline metabolic pathways of fluoride-tolerant strains reached significant levels, among which 3′-adenylic acid and hypoxanthine were significantly upregulated, whereas guanine, allantoic acid, xanthine, N-acetyl-L-glutamic acid, and pyruvate were significantly downregulated. These metabolic pathways may be related to the fluoride tolerance mechanism of NaF poisoning and tolerant strains.

## 1. Introduction

Fluoride is one of the important factors causing environmental pollution. Fluoride-contaminated mulberry leaves are absorbed and accumulated in the midgut of the silkworm after being eaten [1]. The resulting fluorosis has resulted in sericulture farmers incurring huge economic losses, which has seriously damaged the stable development of the sericulture industry. After fluoride poisoning, differences in population development, reduced appetite, and slow production in silkworm are observed. Some internode membranes bulge and appear as ring-shaped spots connected by black spots. Some abdominal links have rough black-brown lesions on the back, which easily rupture and discharge pale yellow blood [2].

The fluoride ion is a protoplasmic toxicant that produces toxicity through the cell wall of mulberry leaves and binds with the protoplasm [3,4]. After eating fluoride-contaminated mulberry leaves, the silkworm accumulates fluoride in the midgut, which then enters the blood and other tissues and organs. The midgut not only serves the function of digesting and absorbing nutrients, it also prevents the invasion of toxic and harmful substances and pathogenic microorganisms. Electron microscopy showed that the structure of the midgut cell layer of the fluorosis silkworm is loose, and many fine particles can be observed in the cytoplasm of the inner wall. During fluorosis, the cell membrane is severely damaged and various organelles are severely degraded [5].

In response to the fluorosis of silkworm, researchers have adopted some control strategies, such as breeding fluoride-tolerant varieties and using drugs [6,7], and many explorations have been conducted into the fluoride-tolerance mechanism at the molecular level. Using fluorescent differential display technology, Zhou et al. [8] obtained *Bmcyp306a1* in the P450 family and another *Bmbcl-2* gene. Real-time fluorescent quantitative PCR analysis showed that the expression of *Bmcyp306a1* in different tissues of fluoride-tolerant and sensitive individuals was significantly different, which might be related to the mechanism of fluoride tolerance. Chen et al. [9] identified five differentially expressed proteins in *Bombyx mori* N cell line (BmN) via two-dimensional electrophoresis. These include two upregulated proteins: mitochondrial aldehyde dehydrogenase (ALDH2) and prohibitin protein WPH, and two upregulated proteins: calreticulin precursor and DNA supercoiling factor. Li et al. [5] found that fluoride stress could inhibit SOD activity and the cholesterol and triglyceride contents, and reduce the content of short-chain fatty acids in the intestinal tract by affecting the water content and pH value of the intestinal contents [7]. A dominant fluoride-tolerant gene was mapped using an SSR marker in our previous research, and 30 upregulated genes were screened from the constructed fluoride-tolerant near-isogenic line (*Def*NIL) by using RNA-seq analysis [2]. The results showed that glutathione metabolism, glycerolipid metabolism, and steroid biosynthesis were the metabolic pathways for significant enrichment of differentially expressed genes (DEGs). These preliminary studies helped to understand the effects of fluoride on some physiological and biochemical indexes of silkworms with different tolerances, and to elucidate the mechanism through which fluoride stress affects silkworm mortality.

Fluoride stress can activate the autoimmune response, including the synthesis of fluoride-tolerant metabolites, or signal molecules to regulate the metabolic process. Metabonomics has been used to further study the functions of some key metabolites by comparing the differences in metabolic patterns between two or more groups of samples. It has been widely used in functional gene research [10,11], metabolic pathway and mechanism analysis [12], and key marker screening [13,14]. There have been reports on the transcriptome [15,16,17], proteome [9], and microbial diversity [5] after NaF stress in silkworm. However, the differences in metabolic pathways of fluoride-tolerant and sensitive silkworm varieties under NaF stress have not been reported using metabonomics.

In this study, metabonomic analysis was conducted using gas chromatography-mass spectrometry (GC-MS) to analyze the changes in midgut of fluoride-tolerant T6 strain and the fluoride-sensitive 733xin strain at the metabolomic level after NaF stress, to screen the differential metabolites, and to further explore the relevant metabolic pathways. Our present study will provide useful information for understanding the fluoride-tolerance mechanism in silkworm.

## 2. Materials and Methods

### 2.1. Silkworm Rearing and Material Treatment

The silkworm was a fluoride-tolerant strain T6 and a fluoride-sensitive strain 733xin preserved in our laboratory. The T6 silkworm strain has high tolerance to fluoride, its larvae can survive with few effects even when they are fed with mulberry leaves treated with 200 mg/Kg of NaF solution. While, the high degree of change in growth in 733xin strain when fed with mulberry leaves even treated with 50 mg/Kg. Fluoride poisoning is a cumulative process. The larvae can recover from early instars fluoride toxicity and convalescence from fluorosis by feeding of fresh fluoride free mulberry leaves in subsequent stages. In order to prevent the early larvae from being too susceptible to poisoning and death, and to facilitate the extraction of tissues, we carried out the present experiment starting from the fourth instar. Silkworms reared to the fourth instar (25 °C, 80% relative humidity) were divided into control and experimental groups. Commercially available anhydrous sodium fluoride (NaF) AR grade produced from Sangon Biotech Ltd., Shanghai, China was used as a toxicant in this research. The control group was fed with fresh mulberry leaves dried naturally after 5 min immersion in clear water, labeled T6_C and 733xin_C, respectively, whereas the experimental group was fed fresh mulberry leaves dried naturally after 5 min immersion in 200 mg/kg NaF solution, labeled T6_F and 733xin_F, respectively. The midgut tissues of different silkworm varieties were dissected and stored in liquid nitrogen. We performed eight replicates per sample.

### 2.2. Sample Preparation

Firstly, put the samples at room temperature after take out from the −80 °C refrigerator, and then added to water of the same volume as the samples. Then, 2 small steel beads were added in turn, placed in the refrigerator at −80 °C for 2 min and ground in a grinder (60 Hz, 2 min). Then, the samples were moved from 100 μL to 1.5 mL tubes. A quantity of 20 μL internal standard (L-2-chlorophenylalanine, 0.3 mg/mL, methanol configuration) was added, followed by whirlpool oscillation for 10 s. Then, 300 μL of methanol-acetonitrile (2:1, *v/v*) mixed solution was added, followed by eddy oscillation for 1 min. After, ultrasonic extraction in an ice water bath was performed for 5 min. After storing for 10 min at −20 °C and centrifuging for 10 min (12,000 rpm, 4 °C), 300 μL supernatant was placed in a glass derivative bottle. Quality control samples (QC) were prepared by mixing the extracts of all samples in equal volume. The volume of each QC was the same as that of the samples. The samples were dried using a freeze-concentration centrifugal dryer. The oxime reaction occurred at 37 °C for 90 min in an oscillating incubator after adding 80 μL methoxyamine hydrochloride pyridine solution (15 mg/mL), followed by swirling oscillation for 2 min. A quantity of 80 μL BSTFA (containing 1% TMCS) derivative reagent and 20 μL hexane was then added to samples. After 2 min of swirl oscillation, the samples were reacted at 70 °C for 60 min. Finally, the samples were collected and placed at room temperature for 30 min for GC-MS metabonomics analysis [18].

### 2.3. GC-MS Analysis Conditions

Analysis was conducted with a 7890B-5977A GC/MSD (Agilent Technologies Inc., San Jose, CA, USA).

The chromatographic conditions were as follows: DB-5MS capillary column (30 m × 0.25 mm × 0.25 μm, Agilent J&W Science, Folsom, CA, USA); carrier gas was high0purity helium (purity not less than 99.999%); flow rate was 1.0 mL/min; and temperature of the inlet was 260 °C. The injection volume was 1 μL not diverted and the solvent was delayed for 5 min. For programmed heating, the initial temperature of the column temperature box was 60 °C, and programmed temperature was 8 °C/min to 125 °C, 5 °C/min to 210 °C, 10 °C/min to 270 °C, and 20 °C/min to 305 °C for 5 min.

The mass spectrometry conditions were as follows: electron bombardment ion source (EI), ion source temperature 230 °C, fourth-stage rod temperature 150 °C, and electron energy 70 eV. Scanning mode was full scan mode (SCAN), with quality scanning range of *m/z* 50 to 500.

A quality control (QC) sample was inserted into every 16 analysis samples to examine the repeatability of the whole analysis process.

### 2.4. Metabonomics Data Preprocessing

The original GC-MS data (D format) was transformed into general format (CDF format) using ChemStation (version E.02.02.1431, Agilent, CA, USA) analysis software, and then imported into Chroma TOF (version 4.34, LECO, St Joseph, MI, USA) software for pre-processing, including peak extraction, noise removal, and deconvolution. Qualitative analysis of metabolites used NIST and Fiehn databases. Finally, peak alignment was performed to derive the three-dimensional (3D) data matrix (original data matrix) in CSV format.

The internal standard was used for data quality control. The internal standard peaks in the original data matrix and any known false positive peaks (including noise, column loss, and derivative reagent peaks) were removed and missing values replaced by 0. In each sample, all peak signal intensities (peak area) were normalized; the relative intensity of each peak signal intensity was transformed into the relative intensity of the spectrum. After data were normalized and multiplied by 10,000, the data matrix was obtained by de-redundancy and peak merging.

### 2.5. Multivariate Statistical Analysis

After log2 conversion (0 was replaced by 0.000001, then converted), the values in the data matrix were imported into the SIMCA software package (version 14.0, Umetrics, Ume, Sweden). Unsupervised principal components analysis (PCA) was used to observe the overall distribution of samples and the stability of the whole analysis process. Then, supervised (orthogonal) partial least squares discriminate analysis (OPLS-DA) was used to distinguish the overall differences of metabolic profiles among groups and to find the different metabolites among groups.

### 2.6. Screening of Differential Metabolites

Multidimensional analysis and one-dimensional analysis were used to screen different metabolites between groups. The criteria for screening were a VIP value of the first principal component (PC1) of the OPLS-DA model > 1 and *p-*value returned by Student’s *t*-test < 0.05. The fold change (FC) of differential metabolites was calculated, in which the change multiple was the ratio of the average content of the differential metabolites in the two groups. FC > 1 indicated that the content of metabolites was upregulated; FC < 1 indicated that the content of metabolites was downregulated.

### 2.7. Metabolic Pathway Analysis of Differential Metabolites

The pathway enrichment analysis of differential metabolites helps with understanding the mechanism of metabolic pathway change in different samples. Common pathway analysis is based on the KEGG metabolic pathway (http://www.genome.jp/KEGG/pathway.html). Mapping differential metabolites to the KEGG database can be used to obtain the enrichment results of their metabolic pathways.

## 3. Results and Analysis

### 3.1. Analysis of GC-MS Results

GC-MS can be used to identify metabolites with low polarity, such as silane derivatives and esters. It provides good coverage of amino acids, organic acids, and carbohydrates [11,19]. Visualization of total ion current (TIC) of all samples showed that, all samples had a strong signal, large peak capacity, and good reproducibility of retention time. The original data of all samples were processed by the analysis software to obtain the data matrix. A total of 1367 peaks were detected, and 484 peaks were detected.

### 3.2. Overall PCA of Metabolic Phenotypes

**Figure 1:**
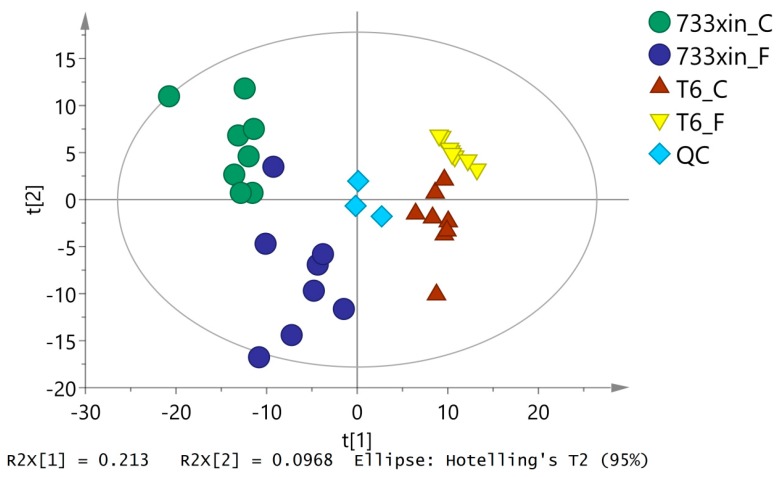
PCA score plots based on the GS-MS data of the *Bombyx mori* midgut samples.

PCA can reflect the overall metabolic differences and the variability in samples between groups as a whole. As shown in Figure 1, the abscissa of the PCA score chart represents the first principal component, PC1, with t [1], and the ordinate represents the second principal component, PC2, with t [2]. The quality control sample is located in the middle, and the machine works well. Samples are relatively concentrated and reproducible, except for eight samples of 733xin_F, which are separated to a certain extent. The unsupervised PCA of all the samples in this experiment showed that R^2^X = 0.48, which indicated that the established model had good fitting accuracy and could be reliably used to explain the metabolic differences between the two groups.

### 3.3. Orthogonal Partial Least Squares Discriminate (OPLS-DA) Analysis

**Figure 2:**
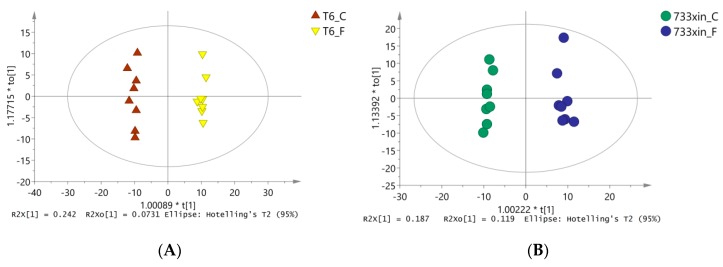

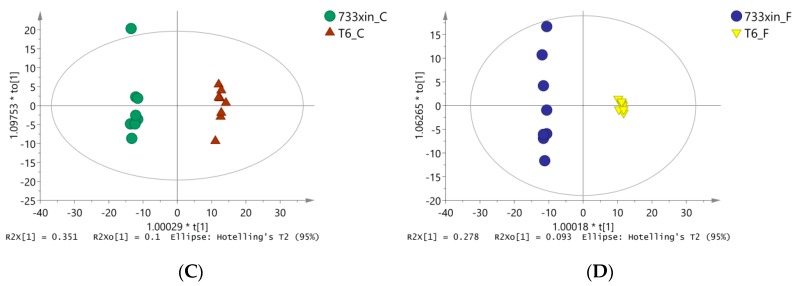
OPLS-DA model diagram. (**A**) OPLS-DA model diagram of T6_F-T6_C; (**B**) OPLS-DA model diagram of 733xin_F-733xin_C; (**C**) OPLS-DA model diagram of T6_C-733xin_C; (**D**) OPLS-DA model diagram of T6_F-733xin_F.Model parameter R^2^Y (the parameter value of this model does not refer to R^2^X value) represents the ability to interpret the model, and Q^2^ represents the ability to predict the model.

To eliminate noise information unrelated to classification (e.g., the control group and processing) and obtain more reliable metabolite information that results in significant differences between the two groups of samples, orthogonal partial least squares discriminate analysis (OPLS-DA) was used to filter the unrelated signals with model classification, i.e., orthogonal, to obtain the OPLS-DA model (Figure 2). We found significant differences (spectral separation) between the two groups on the OPLS-DA score map: R^2^Y = 0.993, Q^2^ = 0.903 between T6_F and T6_C; R^2^Y = 0.989, Q^2^ = 0.805 between 733xin_F and 733xin_C; R^2^Y = 0.996, Q^2^ = 0.967 between T6_C and 733xin_C; and R^2^Y = 0.998, Q^2^ = 0.929 between T6_F and 733xin_F. These four sets of data indicated that the OPLS-DA model has good explanatory and predictive abilities for metabolic phenotypic differences among groups. The 733xin strain has greater metabolic differences after NaF stress, whereas the T6 strain has higher fluoride tolerance, indicating that the metabolic differences after NaF stress are smaller than 733xin.

### 3.4. Screening of Differential Metabolites

A total of 173 differential metabolites were screened and identified between T6_F and T6_C; 70 were upregulated and 103 were downregulated. The differential metabolites included guanine, 3′-adenylic acid, N-acetyl-L-glutamic acid, L-glutamic acid, pyruvic acid, nicotinamide, trehalose, and 3-indoleacetonitrile, indicating that these compounds might be involved in the tolerance reaction of T6 strains to NaF. A total of 136 differential metabolites were screened and identified between 733xin_F to 733xin_C; 75 were upregulated and 61 were downregulated. The differential metabolites included fumaric acid, phosphate, urea, indole-3-acetamide, 2-aminophenol, glycine, and raffinose, suggesting that these compounds might be related to the sensitivity of the 733xin strain to NaF. T6_C–733xin_C screened a total of 209 differential metabolites; 117 were upregulated and 92 were downregulated. We found 182 differential metabolites between T6_F and 733xin_F; 71 were upregulated and 111 downregulated. The greater the VIP value in the OPLS-DA model, the more significant the difference [10,13]. Statistical analysis of the data from the four groups showed that 22 metabolites had significant differences among the four groups (Table 1). These can be classified into carbohydrates, amines and amides, amino acids, fatty acids, organic acids, sterols, bases, etc. Among the four control groups, N-methyl-DL-alanine, phosphate, D-alanyl-D-alanine, and melatonin were significantly upregulated, and 5-hydroxyindole-2-carboxylic acid and cytidine-monophosphate were significantly downregulated.

### 3.5. Metabolic Pathway Analysis of Differential Metabolites

**Figure 3:**
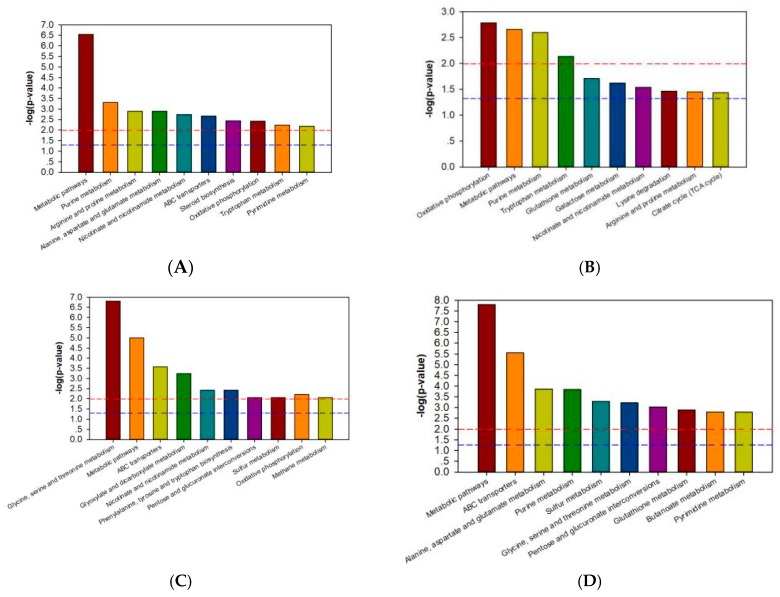
Categories of pathways in which the identified metabolites are involved. (**A**) metabolic pathway enrichment map of T6_F-T6_C; (**B**) metabolic pathway enrichment map of 733xin_F-733xin_C; (**C**) metabolic pathway enrichment map of T6_C-733xin_C; (**D**) metabolic pathway enrichment map of T6_F-733xin_F.

Through the MBRole (http://csbg.cnb.csic.es/mbrole/) pathway analysis function, the KEGG ID of differential metabolites was used for pathway enrichment analysis, and screened differential metabolites were enriched into related pathways. Differential metabolites of 733xin were enriched to 46 metabolic pathways after NaF stress, and differential metabolites of T6 were enriched to 56 metabolic pathways after NaF stress. The metabolic pathway enrichment maps are drawn with the metabolic pathway name as the abscissa and the log (*p*-value) as the ordinate; the maps of the four control groups are shown in Figure 3. In the enrichment map of the metabolic pathway, the red line *p*-value is 0.01 and the blue line *p*-value is 0.05. When the top of the column is higher than the blue line or the red line, the signal pathway it represents significant. The common metabolites of T6 and 733xin under NaF stress include fumaric acid and phosphate. Among the significant metabolic pathways of T6, fumaric acid participates in arginine and proline metabolism; nicotinate and nicotinamide metabolism; oxidative phosphorylation; and alanine, aspartate, and glutamate metabolism. Fumaric acid is involved in oxidative phosphorylation, nicotinate, and nicotinamide metabolism; arginine and proline metabolism; and the TCA cycle in the 733xin metabolic pathway. Phosphate participates in oxidative phosphorylation and ABC transporters in the significant metabolic pathways of T6. It participates in a significant metabolic pathway of 733xin, namely, oxidative phosphorylation (Table 2). The distinct metabolic pathways of T6 are alanine, aspartate, and glutamate metabolism, and ABC transporters, which indicate that the fluoride-tolerant strain T6 effects some metabolic pathway changes through its own unique differential metabolites, thus helping to resist NaF damage. The metabolic pathways of T6 are mainly related to nucleotide metabolism, amino acid metabolism, and energy metabolism.

## 4. Discussion

GC-MS technology has high detection sensitivity and resolution, identifying different metabolites between treatment and control groups. In the present study, OPLS-DA analysis showed that after NaF treatment, the fluoride-sensitive strain showed a clear separation trend. The lower effects of fluoride on T6 strain compared with 733xin strain indicated is lower affinity towards T6, suggesting that the T6 silkworms have tolerance and may metabolize the fluoride to a greater extent than 733xin strain. Pathway enrichment analysis of the screened differential metabolites can help understand the trends in metabolic pathway in different strains.

### 4.1. Energy Metabolism

**Figure 4:**
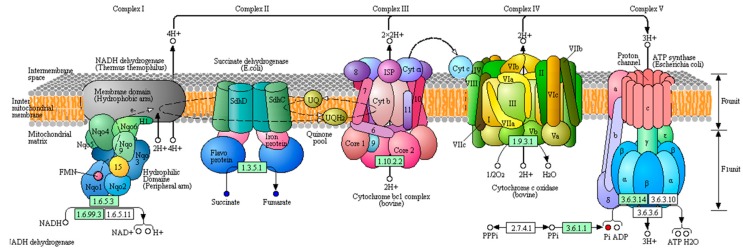
Differential metabolites between T6_F vs. T6_C are located in oxidative phosphorylation. Red dots indicate upregulated in oxidative phosphorylation and blue dots indicates downregulated in oxidative phosphorylation. The originated figure from KEGG pathway database.

Fluoride poisoning in the silkworm is closely related to energy metabolism in tissues and cells [20,21]. Oxidative phosphorylation is the main metabolic pathway through which the energy released during the oxidative decomposition of organic substances is used to produce ATP. The activity of ATPase is related to the secretion of digestive juices and the absorption of nutrients in the midgut tissue [22]. Fluoride can inhibit the activity and affect the distribution of ATPase in the midgut, which results in the obstruction of energy supply and the serious destruction of the absorption function of midgut cells. The main mode of absorption of mesenteric epithelial cells is active transport, which is a process of converse concentration gradient transport that requires a significant amount of energy. The phosphatic content of T6_F–T6_C significantly increased, while fumarate and succinate contents significantly decreased, as shown in Figure 4. The fumarate and phosphate contents significantly increased in 733xin_F–733xin_C. The differential metabolites of the two control groups were enriched in the metabolic pathways, and the results showed that oxidative phosphorylation changed significantly, resulting in abnormal energy metabolism. We speculate that fluoride can act on enzyme block the process of oxidative phosphorylation in the fluoride-sensitive strain 733xin, resulting in an increase in the content of raw materials for ATP production and a decrease in the amount of ATP production. In the fluoride-tolerant strain T6, an oxidative phosphorylation tolerance mechanism and the increase of enzyme activity exist, so the raw materials required for oxidative phosphorylation process are significantly reduced. ABC transports energy from ATP hydrolysis to the plasma membrane, and some members of the ABC family are the main active transporters [23]. This process significantly impacts substance transport, drug tolerance, metabolism, and growth [24]. The ABC transporters metabolic pathway in the T6 strain changed significantly after NaF stress compared with 733xin, suggesting that the significant change in ABC transporters is also related to the fluoride tolerance mechanism of silkworm. Glycometabolism is one of the most important metabolisms in the body. The calcium ion transmits signals from the central nervous system to the organs of the silkworm, and can participate in hormone secretion and other activities. Fluoride ions can combine with calcium and magnesium ions to form more stable compounds, which accumulate in the midgut and exert effects on acid phosphatase, catalase, and other enzymes, thus hindering the normal process of glycometabolism and regulating energy metabolism in the silkworm. This study highlights the significant changes due to the effect of fluoride ion on enzyme in the glucose metabolic pathways in each control group, mainly in the galactose metabolism and TCA cycle of 733xin_F–733xin_C and pentose and glucuronate interconversions of T6_C–733xin_C.

### 4.2. Amino Acid Metabolism

The silkworm excretes uric acid. Excess amino acids produced from food intake or metabolism can be decomposed into carbon shelf structures and ammonia. Most are excreted or accumulated in the epidermis through the malpighian tube in the form of ammonium salts, but small amounts are metabolized by purine compounds to produce uric acid or urea [25,26]. The metabolic pathways of different metabolites in four control groups were enriched and analyzed. We found that amino acid metabolism changed significantly. The melatonin content, a differential metabolite related to tryptophan metabolism between T6_F and T6_C, significantly increased, whereas the 3-indoleacetonitrile, indole-3-acetamide, 2-aminophenol, 5-methoxytryptamine, and 3-hydroxyanthranilic acid contents significantly decreased. The indole-3-acetamide, N-acetyllysine, and melatonin contents, the different metabolites of 733xin_F–733xin_C, significantly increased, whereas the indolelactate and 2-aminophenol contents significantly decreased. Considerable differences exist in the mobilization of metabolites between T6 and 733xin strains. We presume that different tolerance mechanisms to fluoride may be responsible for the differences, and the specific reasons need to be further studied.

Fluoride poisoning can reduce the body’s antioxidant ability. Glutathione is an antioxidant synthesized by glutamic acid, cysteine, and glycine. It can scavenge free radicals and inhibit the initiation and development of peroxidation [27]. Glutathione S-transferases (GSTs) are detoxifying enzymes that widely exist in the midgut of silkworm [28]. They can catalyze the electrophilic group of harmful substances to bind with the sulfhydryl group of reduced glutathione to form more soluble and non-toxic derivatives [29]. That is, GSTs function in detoxification and antioxidation [30,31]. Previous studies showed that increased GST activity in insects can produce tolerance to toxic substances. With the accumulation of NaF concentration in wilkworm, the activity of GSTs in fluoride-tolerance varieties increased, while the acitivity of GSTs in fluoride-susceptible varieties fluctuated greatly, and finally showed a downward trend, which indicated that fluoride was more toxic to sensitive varieties. We speculate that the fluoride tolerance of the silkworm may be related to the GSTs activity [32]. The L-cysteine and glycine contents decreased significantly between 733xin_F and 733xin_C, and L-glutamic acid and putrescine contents decreased significantly between T6F and T6C. The enriched metabolic pathways showed that glutathione metabolism of the 733xin_F–733xin_C group changed significantly. The results showed that fluoride could change the activity of GSTs, which led to a significant decrease in the synthetic enzyme, and finally affected silkworm resistance to fluoride.

**Figure 5:**
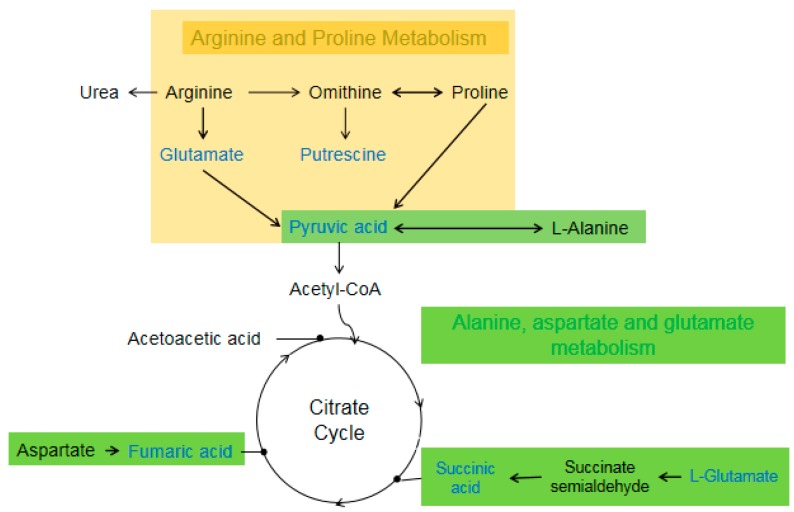
Differential metabolites between T6_F vs. T6_C are located in Arginine, Proline, Alanine, Aspartate, and Glutamate metabolism. Blue color metalites indicates downregulated.

Arginine plays an important role in regulating physiological metabolism and nutrition in silkworm. Arginine produces NO through metabolism; NO is involved in mediating substrate metabolism as a signal molecule [33]. Transamination is an important reaction in amino acid metabolism. Arginine and proline can generate putrescine and pyruvic acid through transamination and enzyme catalysis. Similarly, alanine can also produce pyruvic acid under the action of transaminase. Pyruvic acid can enter the citrate cycle through glycolysis to provide energy for the body. There is a direct deamination of amino acids in insects. Asparate can be directly deaminated to form fumaric acid under the catalysis of asparaginase. Glutamate is finally formed succinic acid by decarboxylase. Both fumaric acid and succinic acid can enter the citrate cycle to oxidize and decompose to provide energy for the body, as shown in Figure 5. After fluoride stress, the contents of fumaric acid, urea, and N-acetyl-L-glutamic acid (the differential metabolites of 733xin_F–733xin_C) increased significantly, whereas the contents of N-acetyl-L-glutamic acid, L-glutamic acid, putrescine, fumaric acid, succinic acid, and pyruvic acid (the differential metabolites of T6F–T6C) decreased significantly. The results of differential metabolite enrichment showed that arginine and proline metabolism in 733xin_F–733xin_C, and arginine, proline, alanine, aspartate, and glutamate metabolism in T6_F–T6_C, underwent significant changes.

### 4.3. Nucleotide Metabolism

**Figure 6:**
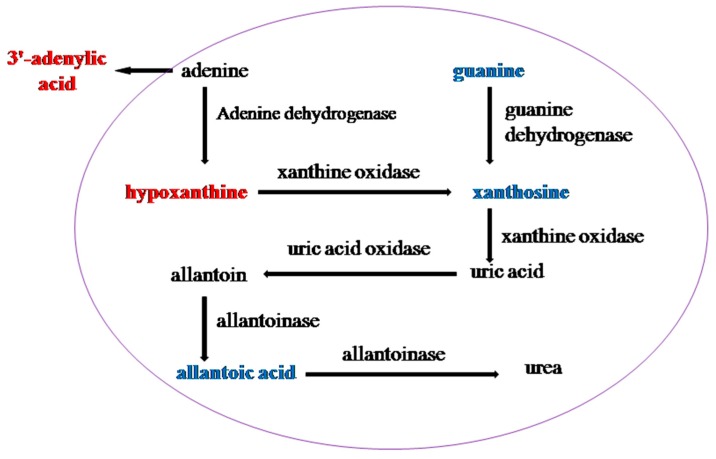
Differential metabolites between T6_F vs. T6_C are located in Purine metabolism. Red indicates upregulated in purine metabolism and blue indicates downregulated in purine metabolism.

Ammonia is extremely toxic to the midgut cells of the silkworm, so it must be removed quickly for detoxification. Uric acid can be produced by purine metabolism in the midgut of the silkworm to excrete excess ammonia. Purine is deaminated by deaminase and hypoxanthine is produced by deamination of adenine. Then, hypoxanthine is oxidized to xanthine by xanthine oxidase, whereas xanthine is directly produced by guanine deaminase and further oxidized to uric acid [34]. The hypoxanthine and 3′-adenylic acid contents, the different metabolites of T6_F–T6_C, significantly increased, whereas the guanine, guanosine, adenosine, xanthine, and allantoic acid contents significantly decreased, as shown in Figure 6. The 3′-adenylic acid and guanine contents, the differential metabolites of 733xin_F–733xin_C, increased significantly, whereas the xanthine content decreased. Purine metabolism enriched by different metabolites changed significantly, and fluoride was able reduce the amount of uric acid excreted by both cultivars, possibly because fluoride can inhibit the conversion of xanthine to uric acid. Pyrimidine metabolism of T6_F–T6_C and T6_F–733xin_F also changed significantly. The uric acid content in the two control groups decreased significantly, indicating that the uric acid production pathway in fluoride-tolerant strain T6 was also inhibited due to the presence of fluoride.

### 4.4. Other Metabolism

Nicotinic acid in the midgut of the silkworm becomes nicotinamide to play a role. Nicotinamide can form coenzyme I and coenzyme II with ribose, phosphoric acid, and adenine. These are coenzymes of many dehydrogenases that dehydrogenate and hydrogenate in the process of biological oxidation. They are closely related to many metabolic processes, including glycolysis, fatty acid metabolism, pyruvate metabolism, and formation of high energy phosphate bonds. The nicotinic acid and fumaric acid contents (the differential metabolites of 733xin_F–733xin_C) significantly increased and nicotinamide was downregulated. The nicotinamide content, the differential metabolite of T6F–T6C, significantly increased, and the nicotinic acid, pyruvic acid, and fumaric acid contents were significantly downregulated. From the changes in differential metabolites, we found that fluoride can inhibit the conversion of nicotinic acid to nicotinamide in the 733xin fluoride-sensitive strain but not in the T6 fluoride-tolerant strain. We speculate that the mechanism of fluoride tolerance in the midgut of the silkworm is related.

## 5. Conclusions

Metabolome analysis showed that both T6 and 733xin silkworm strains induced complex responses under 200 mg/kg NaF stress. The infection of NaF can lead to significant levels of metabolic pathways such as oxidative phosphorylation, purine metabolism, and glutathione metabolism in fluorine-sensitive strain. Among the significantly upregulated differential metabolites are fumaric acid, phosphate and guanine, while the significantly downregulated differential metabolites are L-cysteine, glycine, and xanthosine. It was speculated that fluoride-susceptible strains could respond to NaF infection by changing these metabolic pathways. Metabolic pathways such as purine metabolism, arginine and proline metabolism, ABC transporters, and oxidative phosphorylation of fluoride-tolerant strain were induced changed, and reached a significant level, among which 3′-adenylic acid and hypoxanthine were significantly upregulated, whereas guanine, allantoic acid, xanthine, N-acetyl-L-glutamic acid, pyruvate, fumaric acid, and succinic acid were significantly downregulated. We hypothesized that these metabolic pathways may be related to the fluoride tolerance mechanism of NaF poisoning in tolerant strains. The results can provide some reference for exploring the metabolic mechanism of fluoride regulating the midgut tissue of the silkworm.

## Figures and Tables

**Table 1:** Common differential metabolites in four control groups.

Metabolite	733xin-F vs. 733xin-C			T6-F vs. T6-C			T6-C vs. 733xin-C			T6-F vs. 733xin-F		
	VIP	*p*	FC	VIP	*p*	FC	VIP	*p*	FC	VIP	*p*	FC
raffinose	1.9730	<0.0001	2.6702	1.1615	0.0270	0.7653	1.2283	0.0008	1.9180	1.6267	<0.0001	0.5497
tricetin	1.9949	<0.0001	0.4461	1.1013	0.0379	1.3371	1.2692	0.0008	0.6576	1.4342	0.0009	1.9712
arachidic acid	1.8459	0.0001	0.6691	1.3336	0.0054	1.1608	1.6063	<0.0001	0.4817	1.1006	0.0224	0.8358
androstanediol	1.7021	0.0001	0.6277	1.3489	0.0016	1.3559	1.2781	<0.0001	0.6977	1.2835	0.0010	1.5070
prostaglandin E2	1.7541	0.0005	1.2606	1.1868	0.0145	0.7736	1.0471	0.0082	1.3228	1.3324	0.0018	0.8118
N-methyl-DL-alanine	1.6075	0.0008	1.5624	1.3262	0.0092	1.1077	1.2269	0.0002	1.6073	1.1278	0.0161	1.1396
nicotinamide	1.2384	0.0028	0.5651	1.0728	0.0489	1.0777	1.1909	0.0043	1.2455	1.3247	<0.0001	2.3752
indole-3-acetamide	1.7442	0.0075	2.5556	1.7658	0.0001	0.5501	1.4274	0.0001	1.9831	1.3437	0.0099	0.4269
xylose	1.4202	0.0144	0.3171	1.5321	0.0013	0.4759	1.5303	<0.0001	8.0269	1.7642	<0.0001	12.0456
phosphate	1.3474	0.0149	2.0608	1.4143	0.0063	1.4409	1.3344	0.0002	2.1808	1.0803	0.0229	1.5248
D-alanyl-D-alanine	1.3563	0.0160	1.1455	1.3713	0.0093	1.0976	1.2162	0.0014	1.1518	1.0003	0.0402	1.1036
N-methyl-L-glutamic acid	1.4813	0.0164	2.0773	1.8072	<0.0001	0.4503	1.3420	0.0007	1.7325	1.3931	0.0048	0.3756
glucosaminic acid	1.3853	0.0191	1.3027	1.6018	0.0004	0.7394	1.2018	0.0025	1.3776	1.1969	0.0084	0.7820
guanine	1.4256	0.0207	1.5516	1.6091	0.0010	0.6318	1.1917	0.0091	1.6934	1.4074	0.0014	0.6895
5-methoxyindole-3-acetic acid	1.3218	0.0268	1.4926	1.0875	0.0289	1.4566	1.2071	0.0010	0.5024	1.4787	0.0011	0.4903
stigmasterol	1.2893	0.0412	0.6565	1.2993	0.0055	1.5196	1.2808	0.0015	1.9553	1.7558	<0.0001	4.5257
melatonin	1.1751	0.0416	1.1508	1.3098	0.0166	1.3538	1.2652	0.0010	1.2440	1.3180	0.0061	1.4634
methyl-beta-D-galactopyranoside	1.1474	0.0426	1.7359	1.7925	<0.0001	0.5967	1.0187	<0.0001	8.9987	1.8350	<0.0001	3.0935
alpha-D-glucosamine 1-phosphate	1.2684	0.0449	1.5270	1.8635	<0.0001	0.3464	1.2883	0.0012	1.7887	1.5467	0.0013	0.4058
5-hydroxyindole-2-carboxylic acid	1.8575	0.0041	0.3829	1.4027	0.0182	0.5151	1.3143	0.0051	0.3682	1.8083	<0.0001	0.4954
nicotinic acid	1.4744	0.0064	1.3137	1.7465	<0.0001	0.6829	1.1216	0.0043	0.7923	1.8168	<0.0001	0.4119
cytidine-monophosphate	1.8111	0.0068	0.4009	1.3625	0.0285	0.4958	1.2371	0.0124	0.4089	1.8005	<0.0001	0.5057

The differential metabolites were selected on the basis of the combination of a statistically significant threshold of variable influence on projection (VIP) values obtained from the OPLS-DA model and *p*-values from a two-tailed Student’s *t*-test on the normalized peak areas, where metabolites with VIP values larger than 1 and *p*-values less than 0.05 were included, respectively.

**Table 2:** Partial identified differential metabolites of T6_F vs. T6_C and 733xin_F vs. 733xin_C.

Metabolic Pathway	Metabolite	733xin-F vs. 733xin-C			T6-F vs. T6-C		
		VIP	*p*	FC	VIP	*p*	FC
Purine metabolism	3′-adenylic acid	1.4021	0.0146	1.5897	1.1089	0.0260	1.2742
	guanine	1.4256	0.0207	1.5516	1.6091	0.0010	0.6318
	xanthosine	1.0500	0.0224	0.6018	1.1667	0.0279	0.8304
	guanosine				1.5426	0.0010	0.6137
	allantoic acid				1.4377	0.0034	0.6627
	adenosine				1.4556	0.0038	0.7421
	hypoxanthine				1.4200	0.0048	1.1328
Arginine and proline metabolism	N-acetyl-L-glutamic acid	1.6081	0.0167	1.8397	1.8881	<0.0001	0.3537
	glutamic acid				1.8040	<0.0001	0.7483
	putrescine				1.5495	0.0007	0.8533
	pyruvic acid				1.3513	0.0167	0.5493
	fumaric acid	2.0018	<0.0001	1.2941	1.018	0.0414	0.9117
	Urea	1.7167	0.0004	1.7151			
Alanine, aspartate and glutamate metabolism	L-glutamic acid				1.8040	<0.0001	0.7483
	succinic acid				1.4135	0.0076	0.6709
	pyruvic acid				1.3513	0.0167	0.5493
	fumaric acid	2.0018	<0.0001	1.2941	1.018	0.0414	0.9117
Nicotinate and nicotinamide metabolism	nicotinic acid	1.4744	0.0064	1.3137	1.7465	<0.0001	0.6829
	pyruvic acid				1.3513	0.0167	0.5493
	fumaric acid	2.0018	<0.0001	1.2941	1.018	0.0414	0.9117
	nicotinamide	1.2384	0.0028	0.5651	1.0728	0.0489	1.0777
Oxidative phosphorylation	phosphate	1.3474	0.0149	2.0608	1.4143	0.0063	1.4409
	succinic acid				1.4135	0.0076	0.6709
	fumaric acid	2.0018	<0.0001	1.2941	1.018	0.0414	0.9117
	pyrophosphate	1.5484	0.0054	1.6668			
Tryptophan metabolism	indoleacetonitrile				1.7692	<0.0001	0.4340
	indole-3-acetamide	1.7442	0.0075	2.5556	1.7658	0.0001	0.5501
	aminophenol	1.2944	0.0150	0.0892	1.0252	0.0025	0.4543
	5-methoxytryptamine				1.3455	0.0130	0.7748
	hydroxyanthranilic acid				1.3539	0.0141	<0.0001
	melatonin	1.1751	0.0416	1.1508	1.3098	0.0166	1.3538
	indolelactate	1.7985	0.0013	0.7070			
	N-acetylisatin	1.2651	0.0152	1.4572			
Glutathione metabolism	L-glutamic acid				1.8040	<0.0001	0.7483
	putrescine				1.5495	0.0007	0.8533
	L-cysteine	1.4476	0.0172	0.8256			
	glycine	1.3452	0.0253	0.8108			

The differential metabolites were selected on the basis of the combination of a statistically significant threshold of variable influence on projection (VIP) values obtained from the OPLS-DA model and *p*-values from a two-tailed Student’s *t*-test on the normalized peak areas, where metabolites with VIP values larger than 1 and *p* values less than 0.05 were included, respectively.

## References

[1] Chen Y. (2003). Differences in fluoride effects on fecundity among varieties of the silkworm *Bombyx mori*. Fluoride.

[2] Qian H., Li G., He Q., Zhang H., Xu A. (2016). Analysis of differentially expressed genes between fluoride-sensitive and fluoride-endurable individuals in midgut of silkworm, *Bombyx mori*. Gene.

[3] Anuradha C.D., Kanno S., Hirano S. (2001). Oxidative damage to mitochondria is a preliminary step to caspase-3 activation in fluoride-induced apoptosis in HL-60 cells. Free Radic. Boil. Med..

[4] Zhang M., Wang A., He W., He P., Xu B., Xia T., Chen X., Yang K. (2007). Effects of fluoride on the expression of NCAM, oxidative stress, and apoptosis in primary cultured hippocampal neurons. Toxicology.

[5] Li G.-N., Xia X.-J., Tang W.-C., Zhu Y. (2016). Intestinal microecology associated with fluoride resistance capability of the silkworm (*Bombyx mori* L.). Appl. Microbiol. Biotechnol..

[6] Lin C.Q., Mi Y.D., Yao Q., Wu D.X., Wei Z.J. (1997). The discovery of fluoride resistant dominant gene in silkworm. Sci. Seric..

[7] Chen Y., Du X., Hangzhou Y.J. (2005). Cytochemical evidence for an anomalous dose-response of acid phosphatase activity in the blood but not the midgut of fluoride-treated silkworm larvae, *Bombyx mori*. Fluoride.

[8] Zhou H., Chen K., Yao Q., Gao L., Wang Y. (2008). Molecular cloning of *Bombyx mori* cytochrome P450 gene and its involvement in fluoride resistance. J. Hazard. Mater..

[9] Chen L., Chen H., Yao C., Chang C., Xia H., Zhang C., Zhou Y., Yao Q., Chen K. (2015). The toxicity of NaF on BmN cells and a comparative proteomics approach to identify protein expression changes in cells under NaF-stress: Impact of NaF on BmN cells. J. Hazard. Mater..

[10] Tao H., Li X., Qiu J.-F., Cui W.-Z., Sima Y.-H., Xu S.-Q. (2017). Inhibition of expression of the circadian clock gene Period causes metabolic abnormalities including repression of glycometabolism in *Bombyx mori* cells. Sci. Rep..

[11] Fiehn O., Kopka J., Dörmann P., Altmann T., Trethewey R.N., Willmitzer L. (2000). Metabolite profiling for plant functional genomics. Nat. Biotechnol..

[12] Li Y., Wang X., Chen Q., Hou Y., Xia Q., Zhao P. (2016). Metabolomics Analysis of the Larval Head of the Silkworm, *Bombyx mori*. Int. J. Mol. Sci..

[13] Chen Q., Liu X., Zhao P., Sun Y., Zhao X., Xiong Y., Xu G., Xia Q. (2015). GC/MS-based metabolomic studies reveal key roles of glycine in regulating silk synthesis in silkworm, *Bombyx mori*. Insect Biochem. Mol. Boil..

[14] Wang J., Zhou L., Lei H., Hao F., Liu X., Wang Y., Tang H. (2017). Simultaneous Quantification of Amino Metabolites in Multiple Metabolic Pathways Using Ultra-High Performance Liquid Chromatography with Tandem-mass Spectrometry. Sci. Rep..

[15] Tang W., Zheng X., Li D., Xiao Y., Yang C., Shang S., Shi M., Zhu Y. (2018). Effects of sodium fluoride on the reproductive development of *Bombyx mori*. Environ. Toxicol. Pharmacol..

[16] Tang W., Xiao Y., Li G., Zheng X., Yin Y., Wang L., Zhu Y. (2018). Analysis of digital gene expression profiling in the gonad of male silkworms (*Bombyx mori*) under fluoride stress. Ecotoxicol. Environ. Saf..

[17] Li G., Shi M., Zhao S., Li D., Long Y., Yang C., Zhu Y. (2019). RNA-Seq comparative analysis reveals the response of Enterococcus faecalis TV4 under fluoride exposure. Gene.

[18] O’Hagan S., Dunn W.B., Broadhurst D., Ellis D.I., Brown M., Halsall A., Spasic I., Tseng A., Kell D.B. (2008). A GC-TOF-MS study of the stability of serum and urine metabolomes during the UK Biobank sample collection and preparation protocols. Int. J. Epidemiol..

[19] Patti G.J., Yanes O., Siuzdak G. (2012). Innovation: Metabolomics: The apogee of the omics trilogy. Nat. Rev. Mol. Cell Boil..

[20] Miao Y.G., Jiang L.J., Bharathi D. (2004). Biochemical effects of fluoride on haemolymph of the silkworm, *Bombyx mori* L.. Fluoride.

[21] Chen Z.W., Jia X.Y. (2002). Effects of fluoride on activities of phosphatase in hemolymph and midgut tissues of silkworm. Agro-Environ. Prot..

[22] Li Z., Wang Y., Wang L., Zhou Z. (2018). Molecular and biochemical responses in the midgut of the silkworm, *Bombyx mori*, infected with Nosema bombycis. Parasites Vectors.

[23] Glavinas H., Krajcsi P., Cserepes J., Sarkadi B. (2004). The role of ABC transporters in drug resistance, metabolism and toxicity. Curr. Drug Deliv..

[24] Xie X., Cheng T., Wang G., Duan J., Niu W., Xia Q. (2012). Genome-wide analysis of the ATP-binding cassette (ABC) transporter gene family in the silkworm, *Bombyx mori*. Mol. Boil. Rep..

[25] Hirayama C., Sugimura M., Shinbo H. (1999). Recycling of urea associated with the host plant urease in the silkworm larvae, *Bombyx mori*. J. Insect Physiol..

[26] Tabunoki H. (2016). Can the silkworm (*Bombyx mori*) be used as a human disease model?. Int. Congr. Entomol..

[27] Liang D.G., Tu Z.L., Lu S.L. (2006). Effects of glutathione on oxygen free radicals and malondialdehyde levels in haemolymph of fluorosis silkworm. China Seric..

[28] Nebert D.W., Vasiliou V. (2004). Analysis of the glutathione S-transferase (GST) gene family. Hum. Genom..

[29] Frova C. (2006). Glutathione transferases in the genomics era: New insights and perspectives. Biomol. Eng..

[30] Cheng X., Hu J., Li J., Chen J., Wang H., Mao T., Xue B., Li B. (2018). The silk gland damage and the transcriptional response to detoxifying enzymes-related genes of *Bombyx mori* under phoxim exposure. Chemosphere.

[31] Tan X., Hu X.-M., Zhong X.-W., Chen Q.-M., Xia Q.-Y., Zhao P. (2014). Antenna-Specific Glutathione S-Transferase in Male Silkmoth *Bombyx mori*. Int. J. Mol. Sci..

[32] Li G., Wang X., Weng X., Qian H., Xu A. (2018). Glutathione S-transferase may participate in fluoride resistance in silkworm. Sci. Seric..

[33] Jobgen W.S., Fried S.K., Fu W.J., Meininger C.J., Wu G. (2006). Regulatory role for the arginine–nitric oxide pathway in metabolism of energy substrates. J. Nutr. Biochem..

[34] Fujii T., Banno Y. (2019). Identification of a novel function of the silkworm integument in nitrogen metabolism: Uric acid is synthesized within the epidermal cells in B. mori. Insect Biochem. Mol. Boil..

